# The Effect of Extreme Cold on Complete Blood Count and Biochemical Indicators: A Case Study

**DOI:** 10.3390/ijerph19010424

**Published:** 2021-12-31

**Authors:** Aneta Teległów, Valerjan Romanovski, Beata Skowron, Dawid Mucha, Łukasz Tota, Joanna Rosińczuk, Dariusz Mucha

**Affiliations:** 1 Department of Rehabilitation in Internal Diseases, Institute of Clinical Rehabilitation, Faculty of Motor Rehabilitation, University of Physical Education, 31-571 Krakow, Poland; aneta.teleglow@awf.krakow.pl; 2 Non-Governmental Organization and Association Oswajamy Żywioły, 25-607 Kielce, Poland; valerjanromanovski@gmail.com; 3 Medical Department Diagnostyka S.A., 31-864 Krakow, Poland; beata.skowron1983@gmail.com; 4 Institute of Health Sciences, Podhale State College of Applied Science in Nowy Targ, 34-400 Nowy Targ, Poland; dawid.mucha@ppuz.edu.pl; 5 Department of Physiology and Biochemistry, Institute of Biomedical Sciences, Faculty of Physical Education and Sport, University of Physical Education, 31-571 Krakow, Poland; lukasz.tota@awf.krakow.pl; 6 Department of Nursing and Obstetrics, Division of Internal Medicine Nursing, Faculty of Health Sciences, Wroclaw Medical University, 51-618 Wroclaw, Poland; 7 Department of Biological Regeneration and Correction of Posture Defects, Institute of Biomedical Sciences, Faculty of Physical Education and Sport, University of Physical Education, 31-571 Krakow, Poland; dariusz.mucha@awf.krakow.pl

**Keywords:** cold air, cold water swimming, extreme environment, complete blood count, biochemical profile

## Abstract

Regular exposure to a cold factor—cold water swimming or ice swimming and cold air—results in an increased tolerance to cold due to numerous adaptive mechanisms in humans. Due to the lack of scientific reports on the effects of extremely low outdoor temperatures on the functioning of the human circulatory system, the aim of this study was to evaluate complete blood count and biochemical blood indices in multiple Guinness world record holder Valerjan Romanovski, who was exposed to extremely cold environment from −5 °C to −37 °C for 50 days in Rovaniemi (a city in northern Finland). Valerjan Romanovski proved that humans can function in extremely cold temperatures. Blood from the subject was collected before and after the expedition. The subject was found to have abnormalities for the following blood indices: testosterone increases by 60.14%, RBC decreases by 4.01%, HGB decreases by 3.47%, WBC decreases by 21.53%, neutrocytes decrease by 17.31%, PDW increases by 5.31%, AspAT increases by 52.81%, AlAT increase by 68.75%, CK increases by 8.61%, total cholesterol decreases by 5.88%, HDL increases by 28.18%. Percentage changes in other complete blood count and biochemical indices were within standard limits. Long-term exposure of the subject (50 days) to extreme cold stress had no noticeable negative effect on daily functioning.

## 1. Introduction

The beneficial effects of cold on the human body have been known since ancient times. Cold exposure is one of the strongest physiological and psychological environmental stressors and leads to many significant physiological responses [1,2,3]. The ability to regulate body temperature is among the most important processes for organism survival. In cold environments, the human body adapts to low temperatures through thermoregulation. During the exposure to extremely low temperatures, mechanisms that compensate for heat loss in the body are activated [4].

Cold is a very important factor for humans in nature because by deactivating deep sensory receptors and slowing down the conduction of sensory fibers it is considered the strongest analgesic known to modern medicine. Regular exposure to a cold factor, results in an increased tolerance to cold due to numerous adaptive mechanisms. Swimming in ice-cold water has also been shown to have a positive effect on the mental side of humans [5] and can even be anti-depressive [6].

According to Teległów et al. [7], regular immersion in cold water (winter swimming) increases the deformability of red blood cells in the shrunken blood vessel system after a whole season of winter swimming without accompanying changes in erythrocyte aggregation (aggregation index, amplitude (AMP), total extent of aggregation, half time (T½), kinetics of aggregation, blood pressure variability (BPV), fibrinogen). An increased erythrocyte elasticity in winter swimmers is a kind of protection that facilitates blood cell flow in the shrunken blood vessel system. However, staying too long in an area of reduced temperature causes cell degradation, which can lead to hypothermia [8], pulmonary edema [9,10], and even death. The body’s response to cold involves changes in hormones [11,12], cardiovascular system [13], nervous and muscular systems [14], and immune system [11,12,13,15,16]. There are several mechanisms to prevent hypothermia by constricting skin blood vessels, as well as increasing heat production by intensifying metabolism and the appearance of muscle shivering. When training in low ambient temperatures, the body balances between overheating and cooling. First of all, the muscles work intensely, which enhances heat production. However, the body surface is exposed to low temperatures; the larger the surface area, the faster the body cools. Excessive sweat production or sweat retention between the clothing and the skin promote heat release, which poses a risk when training in negative temperatures because after overheating, when resting, sweat will evaporate, taking heat away from the body and leading to overcooling. In the case of the thermoregulatory mechanisms described above, hypoglycemia inhibits muscle shivering, which limits the body ability to adapt to cold temperatures. It is therefore important to ensure an appropriate balance of dietary fats and carbohydrates when planning an extreme winter workout. Strong, cold wind can increase heat loss. Interestingly, heat loss can be the same at a temperature of −35 °C in an almost windless weather and at a temperature of −25 °C in a 40 km/h wind; the stronger the wind at low temperatures, the greater the heat loss [17].

The purpose of the study was to understand the body’s response to extreme conditions and the capability to adapt to extreme cold.

## 2. Materials and Methods

### 2.1. Subject’s Characteristics

**Figure 1:**
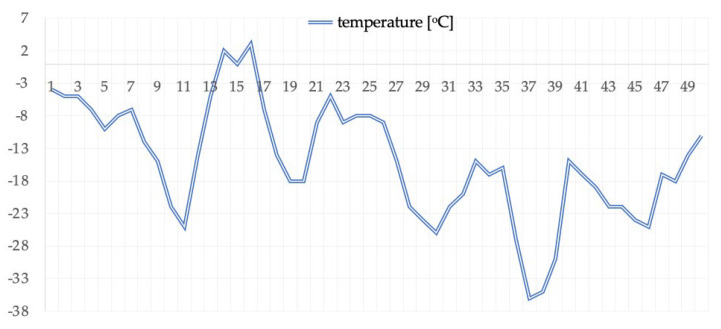
Air temperature changes during the expedition.

The subject of the study is a multiple Guinness World Record holder for low temperatures living in Poland, who was on ice in Rovaniemi at the mouth of the Ounasjoki River to Kemi as part of a research expedition where the air temperature ranged from +2 °C to −37 °C. Air temperature changes throughout the expedition are presented in Figure 1. The premise of the expedition was to survive without external heat sources.

The study subject (age 47) stayed 50 days (late December/early January) in Rovaniemi, northern Finland, at the mouth of the Ounasjoki River to Kemi, a few kilometers south of the northern Arctic Circle (geographic coordinates 66°30′ N, 25°42′ E). The northern parts of Finland, Sweden, Norway, and a small patch of Russia are the Lapland area.

The subject stayed alone, sleeping in an igloo, built on the river, on a 20–30 cm thick ice sheet. While sleeping in the igloo, he insulated himself from the ice with a wooden platform, which was supposed to distance him from the ice surface, and additionally he insulated himself with sheepskin, a sleeping pad, and bubble wrap. The cold protection methods used by the study subject to sleep in an extremely cold environment helped maintain his condition normal. During the expedition, the day lasted 3 h and the night 21 h. He spent 10 h a day in his sleeping bag, and the rest of the time he spent in an active way, covering the route from 5 to 8 h, about 20 km/day, in order not to freeze—that is, walking in the mountains, skiing, and cycling on the ice. He moved all the time to avoid freezing in these low temperatures. His clothing, i.e., overalls, was sewn from goose down, it was worn only in temperatures below −25 °C; in higher temperatures, he used clothing dedicated to survival expeditions (multi-layer clothing). He used footwear that was 2–3 sizes larger, which allowed him to put on a shoe after it had completely ossified; he had a felt insole inside the shoe. The respondent had no hot meals, no campfires, no way to dry clothes. The only source of heat was the energy produced by his body. Throughout the expedition, heart rate was monitored with a Garmin Fenix X6 multisports watch (Finland). Prior to the start of the expedition, the athlete underwent a graded cycling test to determine the physiological indicators at the maximum level and at the second ventilatory threshold level. The heart rate results obtained at the second ventilatory threshold level served as intensity cues for the particular intensities of physical activity undertaken during the expedition. The athlete exhibited a mean resting heart rate of 45.5 ± 4.7 beats/min, mean walking heart rate of 81 ± 5.2 beats/min, mean cycling heart rate of 96.7 ± 6.3 beats/min, mean cross-country skiing heart rate of 109.2 ± 12.5 beats/min, and mean uphill-walking heart rate of 125 ± 12.5 beats/min. The subject’s mean daily energy expenditure (3648.6 kcal) was estimated on the basis of physical activity diaries with the chronometric-tabular method.

Under these extreme conditions, the subject consumed frozen animal protein and fat products, i.e., meat, butter, bread, and cheese, supplemented with vitamin D 2000 IU (1 capsule). He drank 1–2 L water per day, which he obtained by thawing the ice cap with his body, or directly from the river. An excellent mid-day supplement was dried fruit and nuts, which he took with him “on the road”. After intensive expeditions, he usually consumed bee pollen, bee bread, and frozen cheese. Before going to bed he ate honey (he had 7 kinds) with dry bread to warm up his body during sleep. The average daily energy intake was 2223.0 kcal (protein: 83.6 g, fat: 93.5 g, total carbohydrates: 292.5 g), which denotes a moderate food energy density (332 kcal/100 g). The subject kept food diaries throughout the observation period by using an ongoing note-taking method to quantitatively assess the dietary intake. The obtained nutritional data were analyzed with the help of tables of food composition and nutritional value [18]. Mean energy, protein, carbohydrate, and fat intake, as well as energy density per 100 g of food were calculated.

A condition of this research expedition was daily winter swimming in a washing tub, which had a positive effect on his psyche and improved his mood in the dark, as the human body is not adapted to function in such extreme conditions.

### 2.2. Scheme of Study Organization

Before the expedition, somatic, physiological, complete blood count, and biochemical measurements were taken at the University of Physical Education in Krakow (Poland). Immediately after returning from the expedition, somatic, biochemical, and rheological measurements were conducted again.

### 2.3. Somatic Measurements

Body height (BH), body mass (BM), fat mass (FM), percentage body fat (F%), fat-free mass (FFM), body cell mass (BCM), total body water (TBW), and extracellular water (ECW) were measured in anthropometric measurements.

Body mass was determined using a Tanita BC-510 device, body structure was estimated using electrical bioimpedance technique with the AKERN 101 body composition analyzer, and body height was measured using a Martin anthropometer (USA) with a measurement accuracy of 1 mm.

### 2.4. Aerobic Capacity Measurements: Graded Test on a Cycle Ergometer

In order to determine the maximum values of basic physiological indices a “refusal” cycle ergometer test was used. The test effort was started with a four-minute warm-up with a load of 90 W and constant cadence. Then the load was increased by 30 W every 2 min. The aim of the test was to determine the maximum minute oxygen uptake and the level of the second ventilatory threshold (VT2).

During the test, the following indices were recorded using an ergospirometer (Cortex MetaLyzer R3, Leipzig, Germany): minute lung ventilation, percentage carbon dioxide in exhaled air, minute oxygen uptake, minute carbon dioxide excretion, respiratory quotient, and respiratory equivalent for carbon dioxide.

The cycling effort was performed on a cycle ergometer (Monark 834E, Varberg, Sweden), equipped with a timer for the duration of each rotation and connected to a computer. Heart rate during exercise testing was measured using a Finnish-made “Polar S 610 i” device.

### 2.5. Complete Blood Count and Biochemical Measurements

Blood was collected fasting by a qualified nurse from an elbow vein before and after prolonged exposure to extremely cold ambient temperatures. Blood was collected into 2 tubes (Becton Dickinson, Franklin Lakes, NJ, USA) with anticoagulant K2EDTA for complete blood count and 5 mL into a tube without anticoagulant for other biochemical analyses in the Department of Analytics and Clinical Biochemistry, Institute of Oncology, Kraków, Poland.

Complete blood count was performed on an Advia 2120i analyzer (Siemens Healthineers, Erlangen, Germany): RBC [x10^12/L], HGB [g/dL], HCT [%], MCV [fl], MCH [pg], MCHC [g/dL], CHCM [g/dL], RDW [%], HDW [g/dL], HDW [g/dL], WBC [x10^9/L], neutrocytes [x10^9/L], eosinocytes [x10^9/L], basophils [x10^9/L], lymphocytes [x10^9/L], monocytes [x10^9/L], PLT [x10^9/L], MPV [fl], PCT [%], PDW [%].

Coagulation parameters were determined using a BCS Siemens coagulation analyzer: prothrombin activity [%], INR, aPTT [s], fibrinogen [g/L], D-dimer [mg/L].

The following blood biochemical indices were measured in plasma by using a biochemical analyzer Roche/Hitachi Cobas c 501, module 6000: iron [umol/L], B12 [pg/mL], total bilirubin [μmol/L], AspAT [U/L]–aspartate transaminase, AlAT [U/L]–alanine transaminase, GGT [U/L]–gamma-glutamyltransferase, LDH [U/L]–lactate dehydrogenase, total protein [g/L], IgG [g/L], IgA [g/L], IgM [g/L], CK [U/L]–creatine kinase and CK-MB [U/L], cholesterol total [mmol/L], HDL [mmol/L], LDL [mmol/L], TG [mmol/L], alfa-amylase [U/L], lipase [U/L], glucose [mmol/L], HBA1C [%].

Protein electrophoresis was performed on a Sebia Minicap Biameditek analyzer: albumin [g/L], alpha-1-globulin [g/L], alpha-2-globulin [g/L], beta-1-globulin [g/L], beta-2-globulin [g/L], gamma globulin [g/L], AG index. Testosterone [nmol/L], NTproBNP [pg/mL], troponin I [pg/mL], TSH [microIU/mL], FT3 [pmol/L], FT4 [pmol/L] were measured on an Alinity I, Abbott immunochemical analyzer. IL6 [pg/mL] was measured in an Alinity I, Abbott immunochemical analyzer.

### 2.6. Ethical Considerations

The study was approved by the Ethical Committee at the Regional Medical Chamber in Krakow (approval no: 194/KBL/OIL/2019).

### 2.7. Calculation Methods

The results of the observed patient before the expedition were treated as control results. In relation to these, the percentage (%) by which the result changed after prolonged exposure to extremely low temperatures was calculated.

## 3. Results

### 3.1. Somatic Parameters

Table 1 shows selected somatic indices measured before and after the expedition. A decrease in body weight by 9.2 kg (10.8%), fat mass by 5.7 kg (34.2%), and extracellular water by 5.1 L (22.9%) was observed after the 50-day expedition.

Table 2 shows the rates at maximum and threshold levels determined during the graded test. The maximum oxygen uptake was 4.59 L∙min^−1^ (53.7 mL·min^−1^·kg^−1^) and was achieved at a load of 330 W (3.9 W·kg^−1^).

### 3.2. Complete Blood Count

RBC count decreased by 4.01%, HGB value decreased by 3.47%, HCT decreased by 3.40%, RDW decreased by 4.62%, CHCM decreased by 2.10%, MCV increased by 0.64%, MCH increased by 0.33%, HDW increased by 1.21%, MCHC did not change.

WBC count was observed to be decreased by as much as 21.53%, neutrocytes decreased by 17.31%, eosinocytes decreased by 47.37%, basophils decreased by 20%, lymphocytes decreased by 26.85%, and monocytes decreased by 6.90%.

PLT count decreased by 7.50%, MPV decreased by 6.80%, PCT decreased by 14.29%, PDW increased by 5.31%.

### 3.3. Coagulology

Fibrinogen increased by 24.41%, prothrombin activity increased by 19.34%, aPTT increased by 4.67%. Other indices decreased, i.e., D-dimer by 40%, INR by 13.46%.

### 3.4. Blood Biochemical Indices

Liver function tests: We found an increase for AspAT by 52.81, AlAT increase by 68.75%, increase for ALP by 26.35%, and total bilirubin decrease by 40.43%, GGT decrease by 7.69%, LDH decrease by 4.62%.

Immunoglobulins (IgG, IgA, IgM), protein electrophoresis, C-reactive protein: When comparing the result before and after, we found a total protein decrease by 7.03%, albumin decrease by 8.30%, beta-1-globulin decrease by 4%, beta-2-globulin decrease by 10.53%, gamma-globulin decrease [g/L] by 12.30%, A/G decrease by 3.57%, IgA decrease by 9.38, IgG decrease by 7.38%, IgM decrease by 20%, prealbumin decrease by 15.38%. We found an increase for alpha-1-globulin by 8.33%, alpha-2-globulin by 7.55%, C-reactive protein (CRP) by 23.81%

Cardiac profile: We found an increase for CK by 8.61%, CK-MB by 3.10%, troponin I did not change. For NTproBNP, we found a decrease by 32.84%.

Lipid profile: We found an increase for HDL by 28.18%. We observed a decrease for total cholesterol by 5.88%, LDL by 2.04%, TG by 19.78%.

Diabetic profile: We found a decrease for glucose by 11.54%, HBA1C by 9.62%. We observed a decrease for alpha-amylase by 1.30% and an increase for lipase by 23.20%.

Hormonal profile: We found an increase for TSH by 8.33%. For FT3 we observed a decrease by 7.81% and decrease for FT4 26.57%. We observed an increase for testosterone by 60.14% (Table 3).

## 4. Discussion

Short-term physiological stress such as an exposure to cold prepares the immune system to fight infection. But what about long-term exposure to extreme cold? The authors of the presented study are among the first to analyze complete blood count and biochemical changes in blood in humans exposed to extreme temperatures. Based on the available literature and studies published so far, there are few works on this subject.

Blood is a fluid connective tissue that circulates in blood vessels. Through its movement, it ensures close contact between the various organs and cells of the body. Under the influence of environmental factors (extreme cold), changes occur in the microcirculation and in metabolic reactions as well as in the collateral circulation, which can be manifested by changes in the blood.

According to Vogelaere et al. [19], hematological variables are affected by cold stress exposure. According to the authors, cold exposure (0 °C) at rest or during maximal and submaximal physical activity increased the count of RBC, WBC, and PLT, the Hb concentration, and the Ht along with a significant hemoconcentration without affecting MCV, MCH, and MCHC. In the subject presented in this study, RBC count decreased by 4.01%, HGB value decreased by 3.47%, HCT decreased by 3.40%, RDW decreased by 4.62%, CHCM decreased by 2.10%. Based on the analysis of the above results, it can be concluded that this phenomenon was most likely caused by anemia. In the results of the subject, there was a decrease in iron by 26.18% and vitamin B12 by 10.10%. Iron is excreted mainly in urine and to a lesser extent in sweat by people who exercise. In order not to freeze, the subject was walking in the mountains, skiing, and cycling on the ice. Dietary mistakes should also be taken into account (low energy diet combined with high carbohydrate diet often low in easily absorbable iron). It should be mentioned that there is a risk of mechanical trauma, which through regular and prolonged foot-strike hemolysis against the ground in the subject becomes the cause of destruction of red blood cells contained in the blood vessels of the lower limbs. The destruction of erythrocytes is caused by their squeezing through capillaries and the influence of mechanical factors such as an increased acidity, pressure, and body temperature, muscle contractions causing pressure on vessels, oxidative stress, and volume changes in red blood cells. Another cause of post-exercise hemolysis is blood sugar levels that are too low (hypoglycemia). In the subject, there was an 11.54% reduction in glucose, indicating rapid burning of this high-energy compound. There was a slight 0.64% increase in MCV, 0.33% increase in MCH, 1.21% increase in HDW, and no change in MCHC.

Lubkowska et al. [20] demonstrated that cryogenic temperatures affect immune responses, leukocyte mobilization, and cytokine levels. According to the study by Brenner et al. [21], subsequent cold exposure induced a leukocytosis and granulocytosis. Lombardi et al. [22] revealed that winter swimmers are characterized by a significantly higher number leukocytes and platelets subpopulations: neutrophils, lymphocytes, and monocytes. According to these authors, the elevated blood cell counts reflect direct induction of hematopoiesis at the bone marrow level under the pull of sympathetic activity via the activation of a series of hormonal mediators, regulating the energy metabolism and the cold adaptation, and among them, leptin. On the other hand, the subject had a 21.53% decrease in WBC count, 17.31% decrease in neutrocytes, 47.37% decrease in eosinocytes, 20% decrease in basophiles, 26.85% decrease in lymphocytes, 6.90% decrease in monocytes. Leukocytes are blood cells, the so-called guardians of immunity; they are the basic element of a properly functioning immune system. Most likely, the decrease in leukocytes was caused by the subject’s exposure to severe and prolonged stress associated with a drastic change in living conditions and a period-specific diet (frozen food) that was not a source of adequate calories, as well as minerals and vitamins (selenium, zinc, iron, and vitamins A, B, and C) necessary for the proper functioning of the blood marrow-hematopoiesis. Chronic stress reduces the total number of leukocytes and their activity, which was confirmed by the results. It is possible that due to the lowered temperature, the circulation was slowed down and thus the release of leukocytes from the myeloid sinuses into the bloodstream was impaired.

The decrease in WBC count was accompanied by a decrease in PLT count by 7.50%, MPV by 6.80%, PCT by 14.29%, and an increase in PDW by 5.31%. The decrease in PLT count is explained by the decrease in blood viscosity due to the increase in circulating blood volume as a result of adaptive changes in the circulatory system to extreme conditions. Increased PDW with decreased MPV are changes accompanying anemia, which confirms the theory that the subject was anemic in extreme cold, whereas low procalcitonin values are normal values.

It is important to note that the liver is one of the main organs that warms the blood, and therefore the entire body, and this is critical in cold temperatures. The blood that flows out of the liver is about 1 degree Celsius warmer than the blood that reaches it. Under the extreme conditions in which the subject stayed, low air temperature means even below minus 30.2 °C.

Fibrinogen is a protein produced by the liver that participates in clotting; it is a protein involved in hemostasis and is also involved in inflammation and tissue repair. Plasma fibrinogen levels increase 2- to 3-fold during the inflammatory response, which causes cell aggregation and increases blood viscosity [23]. Fibrinogen can modulate the inflammatory response through leukocyte activation and synthesis of pro-inflammatory mediators (cytokines and chemokines) [24,25]. Increased fibrinogen levels are associated with the so-called acute phase responses. Exposure of the subject to extreme cold as a strong stimulus increased fibrinogen by 24.41% (within standard limits). Extreme cold is thought to increase IL-6, a mediator of acute phase response, through chronic stimulation of macrophages. In the subject, IL-6 was elevated by up to 60%. IL-6, through activation of transcription factors of the gene for fibrinogen chain increases its synthesis. IL-6 binds to receptors on the hepatocyte surface and initiates the interaction of nuclear proteins with specific regulatory gene sequences for acute phase proteins [26]. According to Brenner et al. [21], the fall in core body temperature resulting from cold exposure led to a consistent and statistically significant mobilization of circulating cells, an increase in NK cell activity, and elevations in circulating IL-6 concentrations. The adaptation of mechanisms under conditions of an increased heat loss in the immune system is evidenced by the study of Dugue et al. [27], who noted that regular swimming in winter resulted in an increase in interleukins (IL-6) compared with untrained subjects. This is supported by the results obtained by the study of Castellani et al. [28], who observed significant increases in ADH, cortisol, and interleukin-6, which are the most active cytokines involved in immune mechanisms. It is known that IL-6 affects the activity of B and T lymphocytes, bone marrow stem cells, erythroid, and granulocyte-macrophage series cells and stimulates thrombocytopoiesis [29,30]. IL-6 is one of the cytokines included in the so-called positive regulators of hematopoietic stem cell kinetics [31].

Blood clotting is a physiological process that prevents an excessive blood loss due to damage to blood vessels. The subject’s functioning in extreme cold did not impair this process. In the subject, prothrombin activity increased by 19.34%, and aPTT increased by 4.67%. Other indices decreased, i.e., D-dimer by 40%, INR by 13.46% (within standard limits). Low D-dimer concentration allows the body to exclude embolism-thrombosis with high probability, whereas lowering of INR (i.e., increase in coagulability) requires limitation of supply of products rich in vitamin K, i.e., meat and cold meat, which contain the most of this vitamin and which were consumed by the subject for 50 days.

In the states of an increased protein loss, fibrinogen production may increase as a compensatory response to the loss, which was confirmed in the proteinogram indices of the subject, where the following were found within normal limits: total protein decreases by 7.03%, albumin decrease by 8.30%, beta-1-globulin decrease by 4%, beta-2-globulin decrease by 10.53%, gamma-globulin decrease [g/L] by 12.30%, A/G decrease by 3.57%. C-reactive protein is the so-called acute phase protein synthesized in the liver and secreted into the blood. The subject showed an increase by 23.81%, indicating the response of the subject’s body to extreme climatic conditions.

Immunoglobulins play a major role in the humoral immune response and are a heterogeneous group of immune system proteins. Reduced incidence of various diseases in cold-hardened individuals has been linked to higher plasma IgA levels [32]. Janský et al. [32] showed no significant changes after the repeated cold-water immersions for IL-6, immunoglobulins (IgG, IgM, IgA), C-reactive protein. It was concluded that the stress-inducing noninfectious stimuli, such as repeated cold-water immersions, increased metabolic rate due to shivering and the elevated blood catecholamine concentrations of catecholamines, activating the immune system to a slight extent. Banfi et al. [33] studied the effects of systemic cold (cryogenic chamber), inter alia, the immunological parameters of athletes (IgM, IgG, IgA). The obtained results showed no change compared with the baseline measurements. The subject showed a decrease in IgA by 9.38, decrease in IgG by 7.38%, and decrease in IgM by 20%, which may indicate an adaptive process. The decrease in the mean values of the indices of total protein, albumin, globulin, and IgG, IgA, IgM antibodies may indicate the inhibitory effect of extreme cold on the immune system. Most likely, the increase in hormones such as adrenaline and cortisol inhibit the activity of the immune system.

The subject’s liver profile showed significantly elevated AspAT (aspartate aminotransferase) by 52.81% and AlAT (alanine aminotransferase) by 68.75% (above the standard range). Aminotransferase’s activity can be increased in liver lesions but also in cardiovascular lesions. AlAT is an indicator more specific to the liver, while AspAT is also found in heart, muscles, kidney, brain. It is most likely that the diet (frozen fatty meats and processed meats) and the rapid weight loss of 10 kg in 2 months caused hepatic steatosis, which is the accumulation of fat droplets in the liver, which impeded its function and was exposed by an increase in AspAT and AlAT activity or fibrinogen. The theory of hepatic steatosis caused by malnutrition is also supported by the de Ritis index calculated from liver parameters with a value below 1 (AST < ALT). Evaluating the effectiveness of the effect of regular winter swimming on blood biochemical properties, Teległów et al. [2] showed no change due to winter swimming (1–4 °C) on the indices in the liver profile, explaining the long-term adaptation. In turn, other indicators of liver function in the subject were within normal limits: ALP increase by 26.35%, total bilirubin decrease by 40.43%, GGT decrease by 7.69%, LDH decrease by 4.62%. For example, GGT (gamma-glutamyltranspeptidase) activity increases due to consumption of even small amounts of alcohol, which indicates that the subject did not consume alcoholic beverages. Total bilirubin decreases by 40.43%, which is the end product of the breakdown of heme, a compound mainly contained in the hemoglobin of red blood cells (erythrocytes) that was associated with decreased red blood cell and hemoglobin counts in the subject. Low or normal LDH activity usually does not pose a risk, but it can occur in people who take high doses of vitamin C.

In the subject’s cardiac profile, we found an increase for CK by 8.61% and CK-MB by 3.10%, while troponin I did not change. Creatine kinase (CK) activity in the blood increases when skeletal muscle or cardiac muscle cells are damaged. Most likely, the significant increase in CK was caused by skeletal muscle damage after significant exercise, where the subject, in order not to freeze in extremely cold temperatures, was constantly moving about 20 km/day. The changes in the subject’s CK-MB activity were within standard limits, insufficient to classify the event as myocardial damage. The lack of changes in the subject for troponin I is confirmed by the study of Banfi et al. [34], in which the investigators observed that troponin I and hsCRP were unchanged in whole-body cryotherapy (WBC), which consists of exposure to very cold air that is maintained at −110 degrees C to −140 degrees C in special temperature-controlled cryochambers, generally for 2 min. Cardiac markers troponin I and high-sensitivity C-reactive protein, parameters linked to damage and necrosis of cardiac muscular tissue but also to tissue repair, were unchanged, demonstrating that there was no damage, even minimal, in the heart during the treatment.

NTproBNP in the blood is used to assess heart failure; the NTproBNP of the subject decreased by 32.84% (within standard limits). According to Banfi et al. [34], NTproBNP is a parameter linked to heart failure and ventricular power decrease, which shows an increase due to cold stress. However, the NT-proBNP concentrations observed after WBC were lower than those measured after a heavy training session, suggesting that the treatment limited the increase in the parameter that is typical of physical exercise.

The effect of cold temperature on the lipid profile, including LDL concentration, has previously been demonstrated by Ziemann et al. [35] in healthy men after sessions of whole body cryostimulation, and by Lubkowska et al. [20]. In the lipid profile of the subject, we found a decrease in total cholesterol by 5.88%, decrease in TG by 20%, decrease in LDL by 12.34%, and increase in HDL by 28.18%. Low density lipoproteins (LDL) transport cholesterol in the blood. LDL is considered unfavorable because excess LDL causes cholesterol deposition in blood vessel walls, leading to atherosclerosis and ischemic heart disease. LDL is the so-called bad cholesterol. The decrease in total cholesterol, TG, LDL in the subject was due to the high physical activity, for which the body draws energy from the burning of stored fats. Chondronikola et al. [36] demonstrated that the exact effect of non-shivering cold exposure on the modulation of lipid level may be delayed in humans. It should be noted that the physical activity undertaken on a daily basis could also have had a significant impact on the athlete’s lipid profile change. Aerobic-intensity exercise improves cardiovascular fitness regardless of age, gender, or level of physical capacity [37,38,39]. During physical activity, cytokines are released from the muscles, which produce varied metabolic effects on particular tissues [40].

Glucose levels play an important role in adaptation to both cold and exercise. The carbohydrates that the subject consumed mainly in the form of honeys are converted in the liver to glucose, the body’s absorbable sugar. Excess glucose is stored as glycogen or fat. The subject had an 11.54% reduction in glucose (within standard limits), which indicates rapid burning of this high-energy compound. A similar relationship after whole-body cryotherapy (3 min at −110 °C) after 2 months was shown by Teległów et al. [41]. The subject showed a decrease in HBA1C by 9.62%. This combination of glucose and hemoglobin A is called HbA1C, which reflects the average blood glucose concentration over the past months. HbA1C is synthesized and gradually removed from the bloodstream as older red blood cells die off and are replaced by new ones. Amylase is released into the gastrointestinal tract (duodenum) where it is involved in the digestion of carbohydrates-starch and glycogen. We observed a decrease for alpha-amylase by 1.30% (within standard limits), which confirms that there was no damage to pancreatic cells or blockage of pancreatic ducts. We also observed an increase (within standard limits) for lipase by 23.20%. Lipase is an enzyme that catalyzes the hydrolysis of fats to glycerol and fatty acids. Determination of lipase in blood, together with amylase, is helpful in the diagnosis of acute and chronic pancreatitis. In the subject, alpha-amylase and lipase were normal.

When the body is cooled, an increase in thyrotropic hormone (TSH) and thyroid hormone secretion can be observed. This results in an increased metabolism and heat production [42,43,44]. Under extreme cold conditions, non-shivering thermogenesis is a long-term mechanism of heat production, acting through the sympathetic nervous system and thyroid hormones. The maintenance of thyroid function at resting, constant levels depends on the feedback interaction of thyroid hormones with TSH and TRH. Adaptation to changing environmental conditions occurs via TSH. Based on a study by Teległów et al. [41] combining the effects of winter swimming and taking a sauna, no statistically significant changes in the level of TSH, FT3, FT4 were noticed. In the subject, we found an increase for (within standard limits) TSH by 8.33% and FT3 decrease by 7.81% and FT4 decrease by 26.57%. If the concentration of T4 in the bloodstream decreases, the hypothalamus releases thyrotropin-releasing hormone, which in turn stimulates the secretion of thyrotropin hormone (TSH) by the pituitary. TSH stimulates the production and release of more T4. If, due to thyroid disorders or insufficient TSH levels, the thyroid gland does not produce sufficient amounts of T4, symptoms of hypothyroidism such as weight gain, dry skin, cold intolerance, and fatigue occur. In the study subject, no such symptoms occurred; his body tolerated extreme cold for 50 days.

Testosterone stimulates protein assimilation and growth of muscles. Testosterone administration (T) increases lean body mass and muscle protein synthesis [45]. Sakamoto et al. [46] suggest that physical exercise (a bicycle ergometer load) increases TS level in serum by increasing LH and NA levels, but these tendencies were not found with cold water stimulation. The testosterone concentration in the subject increased by 60.14%, which is an important example of how the body fights for survival of the species under extreme conditions. It is the most important male sex hormone. The increase in testosterone levels confirms that the body’s living conditions have significantly worsened and the body has set its sights on survival. The subject spent his time actively, walking about 20 km every day to avoid freezing (walking in the mountains, skiing, and cycling on the ice), which also increased testosterone levels, according to the report by Fujibayashi et al. [47], who demonstrated that TS increased rapidly in response to the accommodation to height in mountain climbing, or Ismail et al. [48], who implied that periodical physical exercise increased TS in hard physical exercise such as marathon running.

## 5. Conclusions

The stimulating factors in this study included not only extreme cold but also physical activity and methods of keeping warm. The cold protection methods used by the study subject to sleep in an extremely cold environment helped maintain his condition as normal; people staying in extremely cold conditions must apply cold protection methods to sleep. The subject’s testosterone concentration increased by 60.14%, which is an important example of how an organism fights for survival of the species under extreme conditions. The subject’s RBC count decreased by 4.01% and HGB value by 3.47%, which indicates anemia, as the subject also had a decrease in iron by 26.18% and vitamin B12 by 10.10%, which indicates dietary errors. The 21.53% decrease in WBC count and 17.31% decrease in neutrocytes were observed in the subject due to exposure to severe and prolonged stress, associated with a drastic change in living conditions, as well as the diet specific for this period (frozen food), which was not a source of an adequate number of calories, minerals, and vitamins (selenium, zinc, iron, and vitamins A, B, and C) necessary for the proper functioning of blood marrow-hematopoiesis. Chronic stress decreases the total number of leukocytes and impairs their activity, as confirmed by the results. Elevated PDW with decreased MPV are changes associated with anemia, which supports the theory that the subject became anemic under extreme cold conditions. The liver profile showed significantly elevated AspAT (aspartate aminotransferase) by 52.81% and AlAT (alanine aminotransferase) by 68.75% (above the standard range). Most likely, the subject’s diet (frozen fatty meats and processed meats) and rapid weight loss of 10 kg in 2 months caused hepatic steatosis. The theory of hepatic steatosis caused by malnutrition is also supported by the de Ritis index calculated from liver parameters with a value below 1 (AST < ALT). The increase in CK by 8.61% above the normal range was caused by skeletal muscle damage after significant physical activity, where the subject, in order not to freeze in extremely low temperatures, kept moving about 20 km/day. It should also be emphasized that the physical activity undertaken on a daily basis could also have had a significant effect on the change in the subject’s lipid profile.

## Figures and Tables

**Table 1:** Anthropometric indices.

Index → Measurement ↓	BM [kg]	BCM [kg]	FFM [kg]	FM [kg]	F% [%]	TBW [L]	ECW [L]
Measurement I	85.5	41.2	65.9	19.6	23.0	51.4	22.3
Measurement II	76.3	36.0	62.4	13.9	17.8	44.1	17.2

Abbreviations: BM, body mass; BCM, body cell mass; FFM, fat-free mass; FM, fat mass; F%, body fat percentage; TBW, total body water; ECW, extracellular water.

**Table 2:** Exercise (threshold and maximum) levels of selected physiological indices.

Parameters	t [min]	P [W]	HR [Beats/min]	VO_2_ (L∙min^−1^)	VO_2_/kg (mL·min^−1^·kg^−1^)	Ve [L/min]
VT2	14.00	225	138	3.24	37.9	78.4
max	21.00	330	159	4.59	53.7	132.3

Abbreviations: t, test duration; P, load; HR, heart rate; VO_2_, maximum minute oxygen uptake globally; VO_2_max, maximum minute oxygen uptake relative to body weight; VE, minute lung ventilation.

**Table 3:** Patient outcomes before and after prolonged exposure to extreme cold.

Analyzed Parameter (Unit of Measure)	Results before Exposure to Extreme Cold	Results after Exposure to Extreme Cold	Change Compared to the Result before (% of Change)	Standard
RBC [×10^12/L]	4.74	4.55	−4.01	4.6–6.2
HGB [g/dL]	14.4	13.9	−3.47	14–18
HCT [%]	44.1	42.6	−3.40	42–52
MCV [fl]	93.1	93.7	0.64	82–94
MCH [pg]	30.5	30.6	0.33	27–32
MCHC [g/dL]	32.7	32.7	no change	30–38
CHCM [g/dL]	33.3	32.6	−2.10	31–37
RDW [%]	13	12.4	−4.62	11.5–14.5
HDW [g/dL]	2.48	2.51	1.21	2.2–3.2
WBC [×10^9/L]	4.18	3.28	−21.53	4–10
Neutrocytes [×10^9/L]	2.08	1.72	−17.31	2.2–7.0
Eosinocytes [×10^9/L]	0.19	0.1	−47.37	0.0–0.5
Basophils [×10^9/L]	0.05	0.04	−20.00	0.0–0.1
Lymphocytes [×10^9/L]	1.49	1.09	−26.85	1.0–3.5
Monocytes [×10^9/L]	0.29	0.27	−6.90	0.0–0.9
PLT [×10^9/L]	200	185	−7.50	150–400
MPV [fl]	10.3	9.6	−6.80	7.2–11.1
PCT [%]	0.21	0.18	−14.29	0.120–0.380
PDW [%]	65.9	69.4	5.31	28.0–60.0
**Coagulology**				
Prothrombin activity [%]	92.17	110	19.34	80–130
INR	1.04	0.9	−13.46	0.85–1.15
aPTT [s]	25.7	26.9	4.67	20.0–36.0
Fibrinogen [g/L]	2.13	2.65	24.41	1.8–3.5
D-dimer [mg/L]	0.17	0.102	−40.00	<0.5
**Biochemistry**				
Iron [umol/L]	23.3	17.2	−26.18	14.0–32.0
B12 [pg/mL]	406	365	−10.10	200–900
Total bilirubin [umol/L]	9.4	5.6	−40.43	2.0–21.0
ALP [U/L]	44.4	56.1	26.35	
AspAT [U/L]	26.7	40.8	52.81	5–38
AlAT [U/L]	30.4	51.3	68.75	5–41
GGT [U/L]	13	12	−7.69	5–55
LDH [U/L]	179.6	171.3	−4.62	100–225
Total protein [g/L]	76.8	71.4	−7.03	64–83
Albumin [g/L]	48.2	44.2	−8.30	38.0–49.0
Alpha-1-globulins [g/L]	2.4	2.6	8.33	2.4–4.0
Alpha-2-globulins [g/L]	5.3	5.7	7.55	4.8–9.0
Beta-1-globulins [g/L]	5	4.8	−4.00	3.2–6.3
Beta-2-globulins [g/L]	3.8	3.4	−10.53	2.2–5.2
Gamma globulins [g/L]	12.2	10.7	−12.30	6.8–15.0
AG index	1.68	1.62	−3.57	
IgG [g/L]	12.2	11.3	−7.38	6.9–14.0
IgA [g/L]	3.2	2.9	−9.38	0.9–4.1
IgM [g/L]	1.5	1.2	−20.00	0.3–2.1
CRP [mg/L]	0.21	0.26	23.81	<5.0
Prealbumins [g/L]	0.377	0.319	−15.38	0.210–0.410
CK [U/L]	239.3	259.9	8.61	24–198
CK-MB [U/L]	12.9	13.3	3.10	4–24
Total cholesterol [mmol/L]	6.29	5.92	−5.88	3.0–5.0
HDL [mmol/L]	1.1	1.41	28.18	>1.0
LDL [mmol/L]	4.9	4.31	−12.04	<3.0
TG [mmol/L]	1.82	1.46	−19.78	<1.7
Alpha-amylase [U/L]	30.7	30.3	−1.30	28–100
Lipase [U/L]	25	30.8	23.20	13–60
Glucose [mmol/L]	5.89	5.21	−11.54	3.9–5.5
HBA1C [%]	5.2	4.7	−9.62	4.0–6.5
**Immunochemistry**				
Testosterone [nmol/L]	23.33	37.36	60.14	4.94–32.01
NTproBNP [pg/mL]	33.5	22.5	−32.84	≤125
Troponin I [pg/mL]	10	10	no change	0.26.2
TSH [microIU/mL]	0.72	0.78	8.33	0.35–4.94
FT3 [pmol/L]	5.76	5.31	−7.81	2.63–5.70
FT4 [pmol/L]	15.58	11.44	−26.57	9.0–19.0
IL6 [pg/mL]	1.5	2.4	60.00	≤7.0

Abbreviations: RBC, red blood cell; HGB, hemoglobin; HCT, hematocrit; MCV, mean corpuscular volume; MCH, mean corpuscular hemoglobin; MCHC, mean corpuscular hemoglobin concentration; CHCM, cellular hemoglobin concentration mean; RDW, red cell distribution width; HDW, hemoglobin distribution width; WBC, white blood cell; PLT, platelet; MPV, mean platelet volume; PCT, procalcitonin; PDW platelet distribution width; INR, international normalized ratio; aPTT, activated partial thromboplastin time; ALP, alkaline phosphatase; AspAT, aspartate aminotransferase; AlAT, alanine aminotransferase; GGT, gamma-glutamyltranspeptidase; LDH, lactate dehydrogenase; IgG, immunoglobulin G; IgA, immunoglobulin A; IgM, immunoglobulin M; CRP, C-reactive protein; CK, creatine kinase; CK-MB, creatine kinase MB, cardiac marker; HDL, high-density lipoprotein; LDL, low-density lipoprotein; TG, triglyceride; HBA1C, glycohemoglobin; NTproBNP, B-type natriuretic peptide; TSH, thyroid-stimulating hormone; FT3, free triiodothyronine; FT4, free thyroxine; IL-6, interleukin 6.

## Data Availability

The authors confirm that all data underlying the findings described in this manuscript are fully available to all interested researchers upon request.
